# Actual Body Weight and the Parent’s Perspective of Child’s Body Weight among Rural Canadian Children

**DOI:** 10.3390/children3030013

**Published:** 2016-08-04

**Authors:** Chandima P. Karunanayake, Donna C. Rennie, Carole Hildebrand, Joshua A. Lawson, Louise Hagel, James A. Dosman, Punam Pahwa, the Saskatchewan Rural Health Study Team

**Affiliations:** 1 Canadian Centre for Health and Safety in Agriculture, University of Saskatchewan, Box 23, 104, Clinic Place, Saskatoon, SK S7N 2Z4, Canada; donna.rennie@usask.ca (D.C.R.); clh631@shaw.ca (C.H.); jal226@mail.usask.ca (J.A.L.); louise.hagel@usask.ca (L.H.); james.dosman@usask.ca (J.A.D.); pup165@mail.usask.ca (P.P.); saskatchewan.rural@usask.ca; 2 College of Nursing, University of Saskatchewan, 104 Clinic Place, Saskatoon, SK S7N 2Z4, Canada; 3 Department of Medicine, College of Medicine, University of Saskatchewan, 5D40 Health Sciences Building, Box 19, 107 Wiggins Road, Saskatoon, SK S7N 5E5, Canada; 4 Department of Community Health and Epidemiology, University of Saskatchewan, Health Science Building, 104, Clinic Place, Saskatoon, SK S7N 5E5, Canada

**Keywords:** rural, body weight, body mass index

## Abstract

The prevalence of being overweight during childhood continues to increase in the USA and Canada and children living in rural areas are more at risk than their urban counterparts. The objectives of this study were to evaluate how well the parent’s perception of their child’s weight status correlated with objectively measured weight status among a group of rural children and to identify predictors of inaccurate parental perceptions of child’s weight status. Participants were children from the Saskatchewan Rural Health Study conducted in 2010. Self-administered questionnaires were distributed through rural schools to parents of children in grades one to eight. Parents reported their child’s height and weight and rated their child’s weight status (underweight, just about the right weight, or overweight). Standardized body mass index (BMI) categories were calculated for clinically measured height and weight and for parental report of height and weight for 584 children. Logistic regression analysis was performed to identify predictors of misclassification of the parent’s perception of child’s weight status adjusting for potential confounders. Clinically measured overweight was much higher (26.5%) compared to parental perceived overweight (7.9%). The misclassification of the child’s BMI was more likely to occur if the child was a boy (odds ratio (OR) = 1.58) or non-Caucasian (OR = 2.03). Overweight was high in this group of rural children and parental perception of weight status underestimated the actual weight status of overweight school-age children. Parental reporting of child weight status has implications for public health policy and prevention strategies. Future research should focus on assessing longitudinal effects of parental misperceptions of child’s weight status.

## 1. Introduction

Over the past three decades, the prevalence of childhood obesity has continued to increase in the USA and Canada [1,2,3,4,5,6]. According to Statistics Canada (http://www.statcan.gc.ca/eng/start), data from the 2009–2011 Canadian Health Measures Survey indicates that 19.8% of children (5–17 years old) were overweight and 11.7% were obese [7]. Previous Canadian studies [8,9,10,11] and USA research [12] reported that rural children are at a higher risk of being overweight or obese compared to their urban counterparts. Plotnikoff et al. reported that the prevalence of overweight was higher in rural boys than urban boys (17.6% versus 12.4%) while the prevalence of obesity was higher among rural girls than urban girls (4.8% versus 2.3%) [9]. Bruner and others observed that the Canadian adolescents living in rural areas were more likely to be overweight and obese compared to urban adolescents [11]. In a recent Canadian study of rural Saskatchewan, the prevalence of being overweight and obese was reported at 25.5% and 7.1%, respectively, indicating that overweight rates are high among rural children [10].

There are many health consequences due to childhood obesity. It can be linked to several medical conditions such as fatty liver disease, sleep apnea, cardiovascular disease and type 2 diabetes [13]. Obesity in childhood can affect a child’s social and emotional health and their academic performance [13]. Also, overweight and obese children are more likely to continue to be overweight in adulthood [7,14,15]. Parents’ perspective of their child’s weight has been shown to play an important role in the long-term health of the child and has implications for addressing preventive strategies, such as an appropriate diet and exercise to maintain or attain a healthy weight [16,17,18,19].

Previous studies have reported that parental perception of their child’s weight is different from the measured weight status using the body mass index (BMI) category scale [15,16,20,21,22]. While studies have shown discrepancies between clinical data versus parental perceptions of their child’s weight or parental reported weight and height [23], only a few Canadian studies have reported findings that examine either parents’ perceptions or reports of their child’s weight in relation to clinically measured BMI for their children [16,21,24].

There are limited data available that imparts parents’ perception of their child’s weight for rural Canadian children. The primary objective of this study was to evaluate how well the parent’s perception of their child’s weight status correlated with objectively measured weight status among a group of rural children. Another objective was to identify predictors of inaccurate parental perception of their child’s weight status.

## 2. Materials and Methods

### 2.1. Subjects and Procedure

The study participants were children from the Saskatchewan Rural Health Study (SRHS)—Child Cohort [25,26]. After approval from the school district boards and principals, questionnaires were distributed to parents of SRHS children in grades 1 to 12 (6 to 17 years of age) in four selected quadrants in rural Saskatchewan through the schools. Forty-three schools within the same rural municipalities used for the adult portion of the SRHS [27] were chosen to collect information on children. Of the 43 selected schools, 39 of these agreed to participate. Parents were asked to respond to the inquiries about their child’s health and return the questionnaires to the school classroom. A total of 5667 study packages were distributed, 2757 (49%) were returned, with complete questionnaires from 2383 (42%) participants and 374 (13.6% of return study packages) refusing to take part. In order to reduce costs and maximize the efficiencies of data collection, researchers selected 1748 children in grades 1 to 8 (6 to 14 years of age) in 16 schools for clinical testing based on response rates within schools during the questionnaire elements of the study. Of those 1768 children invited to take part, 626 (35.4%) children provided written assent and parental written consent for the clinical assessment. Clinical assessments were completed by 584 children. Anthropometric measurements (abdominal girth, height and weight) were obtained. Height was measured against a wall using a fixed tape measure with subjects standing in socks on a hard floor. Weight was measured using a calibrated spring scale with subjects in socks and dressed in indoor clothing. Using clinical measures of weight and height, BMI was calculated based on the equation of BMI = weight (kg)/height (m)^2^ [28].

### 2.2. Questionnaire Data

Questionnaire data was also collected from parents on the children’s reported height, weight, age, sex, ethnicity (Caucasian or non-Caucasian), the child’s current and past health history, difficulty of obtaining routine or on-going health care for the child, the frequency of different foods in child’s weekly diet, their physical activity level (number of days/week and recent activity), type of household (single parent or two parents/partner), home location (farm or non-farm), person completing the questionnaire and parents’ level of education.

#### Parental Perceptions

Parents’ perception of their child’s weight status was rated using the question ‘Do you consider your child to be (underweight, just about the right weight, overweight)?’ This inquiry was from a question used in the National Health and Nutrition Examination Survey III [29].

### 2.3. Classification of Overweight and Obese

BMI categories (underweight, normal, overweight or obese) were derived according to age/sex-specific classification cut-offs established by the International Obesity Task Force [30,31] for clinical measures of weight and height and the parental report of weight and height. Overweight and obese categories of clinically measured BMI (calculated using measured weight and height) and parental report of BMI (calculated using reported weight and height) were combined to compare with the parent’s reported perception of BMI categories (underweight, just about the right weight or overweight).

### 2.4. Statistical Analysis

A new binary variable (yes/no) was created to identify the misclassification of BMI by the parent’s perception of the child’s weight based on the clinically measured BMI. Misclassification was ‘yes’ when parents incorrectly classified their overweight child as normal weight or underweight; or incorrectly classified their normal weight child as underweight or overweight; or incorrectly classified their underweight child as normal weight or overweight.

Statistical analyses were conducted using SPSS (IBM SPSS Statistics for Windows, Version 22.0, IBM Corp, Armonk, NY, USA, 2013). Descriptive analyses were completed using frequencies, measures of central tendency, variability and chi-square test of associations. Logistic regression models were used to predict the relationship between the binary outcome of misclassification of BMI (yes or no) and a set of predictor variables (child’s age, sex, ethnicity, presence of any health conditions, difficulty of obtaining routine or on-going health care, fast food/drinks intake, physical activity, type of household, home location, person completing the questionnaire and parents’ highest level of education) based on their clinical importance reported in previous studies. Based on the bi-variable analysis, variables with *p* < 0.20 became candidates for a multivariable model. All variables that were statistically significant (*p* < 0.05), as well as important covariates, were retained in the final multivariable model. A parsimonious model was selected based on the Hosmer-Lemeshow goodness-of-fit statistic [32]. The strengths of associations were presented as odds ratios (ORs) and their 95% confidence intervals (CIs).

## 3. Results

There were 584 children with parental report of the questionnaire survey and clinical assessments. Of those, 307 were boys (52.6%) and 277 were girls (47.4%). The average age was approximately 10 years (standard deviation (SD) = 2.2) with the majority of children reported to be Caucasian (90.4%). There were 7.9% children who were perceived as being overweight by their parent. The prevalence of being overweight based on clinical measurements, was much higher (26.5%) in comparison to the parent’s perception (Table 1).

Table 2 shows the association between parental perception of their child’s weight status and parental report of their child’s weight status with the child’s measured weight status at the time of the clinical assessment. The majority of parents (93.8%) correctly perceived their child as being just about the right weight. On the other hand, 71% of parents of overweight children classified by clinical measures, perceived that their child was just about the right weight, indicating that, many parents of overweight children inaccurately perceived their child’s weight status. Compared to the child’s actual measured weight, the classification of weight status based on parental report of weight status appeared to be more accurate than parental perception of their child’s weight status for underweight or overweight (underweight: 59.1% versus 39.3%; overweight: 77.3% versus 29.0%) children. However, this was not the case for normal weight children where parental perceptions more correctly identified normal (just about the right weight) weight status compared to parental report of normal weight status (see Table 2). Accurate perception of weight status in the total sample was 74%. Accurate weight status reported in the total sample was 80%. Overall, the accuracy of the parent reported weight was higher than the parental perception of weight status.

Table 3 shows the comparison of the parental report and actual measurement of weight, height and calculated BMI stratified by sex. There was a significant underestimation of weight and height for both boys and girls. There was no significant difference between parent reported and clinically derived BMI for both boys and girls.

**Figure 1:**
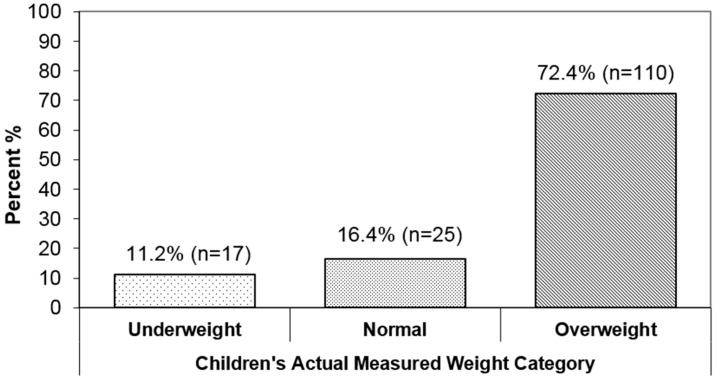
Distribution of misclassification of parent’s perceived weight status in rural Saskatchewan children.

About 26.0% of children (152/584) were misclassified to underweight, normal or overweight categories. Figure 1 displays the misclassification categories. Of those misclassified, 72.4% were from the overweight group, 11.2% were from the underweight group and 16.4% were normal weight children (Figure 1). We observed that all parents of misclassified overweight children were underestimating their child’s weight. In contrast, all parents of misclassified underweight children were overestimating their child’s weight. Of the parents of misclassified normal weight children, 96% were underestimated and only 4% were overestimated. The misclassification was higher for boys (28.7%) compared to girls (23.1%). When stratified by sex, underestimation was higher for boys (88.6%) compared to girls (87.5%) and not statistically significant (data not shown).

Logistic regression analysis was performed to identify the predictors of the misclassification of parent’s perceived weight status based on clinical measurements. Unadjusted and adjusted logistic regression results are presented in Table 4. Male gender (OR = 1.58) and from a non-Caucasian ethnic background (OR = 2.03) were more likely to misclassify a child’s weight status using the BMI scale adjusting for other covariates (Table 4). The Hosmer and Lemeshow Test (χ^2^ = 12.09, degrees of freedom = 8; *p*-value = 0.147) indicates that numbers of misclassification are not significantly different from those predicted by the model and the overall model fit is good. There were no significant associations between the misclassification of parent’s perceived weight status and other factors (Table 4). A sub analysis was conducted only with overweight children (*n* = 155) and observed similar associations with all variables except for ethnic background (data not shown).

## 4. Discussion

This study shows that correctly recognizing children’s overweight or obese status was a problem for rural parents. It is observed that more parents tend to accurately report their child’s weight status when reporting height and weight separately than when categorising their child’s weight into underweight, normal weight or overweight. Overall, 26% of parents were not able to accurately identify their children’s weight categories. Of the 26% of misclassified, 72.4% of overweight children’s weights were underestimated. Parents were more likely to misclassify their child’s weight status if the child was a boy and from a non-Caucasian ethnic background.

Although parental report of weight, height and calculated BMI were underestimated with respect to the clinically measured weight, height and calculated BMI, mean differences were small. Similar to this study, Scholtens reported that the mean weight, height and BMI reported by parents corresponded well with measured values [33]. Powell-Young found that parental report of children’s weight was underestimated and children’s height was overestimated in comparison to the actual measured weight and height, resulting in an underestimation of BMI based on parental report [33]. Still, this author suggests the parent report of their children’s height and weight may be useful information in studies, particularly in large scale cohorts, where the parental report of weight and height were easily obtainable [34].

Many studies reported that parents do not correctly identify their children’s weight status with the percentage of misclassification ranging from 20% to 80% [3,15,16,19,21,29,35,36,37,38,39,40,41,42,43,44,45]. In a Canadian study, 63% of parents classified their children as normal weight when in fact, they were overweight [24]. Results from a recent systematic review of mothers’ perceptions of the nutritional status of their overweight children demonstrated that mothers tend to underestimate children’s overweight and obesity status [46]. The ability of parents to recognise their child’s overweight or obese status according to an internationally recommended standard may be limited [37]. It is possible that parents do not have a clear definition of what overweight means and they might gauge their child’s health by their activity level rather than their weight [45]. Also, parents tend not to believe in physician/clinical charting of weight and BMI percentiles [47]. Jones et al. noted that a common approach by parents in estimating overweight in children was to visually compare their children with others [37].

The misclassification of children’s weight is consistent across countries [45,48]. A systematic review of parents’ ability to identify overweight children based on internationally recognised standards found that, in 19 out of the 23 studies that were examined, less than 50% of parents failed to classify their children as overweight when they should have been so classified [48]. Parents inaccurately identifying their child’s weight has the potential to lead to a steady increase of weight, not having the ability to support weight loss measures, and the children having possible health issues in their adult life [45].

Several factors are known to be associated with parental misclassification of weight status of a child. The age of a child possibly influences a parent’s recognition of the child’s actual weight status [20,49]. Even though we did not observe a statistically significant association between child’s age and the misclassification of weight status, there was a trend for misclassification of weight with older children. Similarly, Maynard et al. reported that older children were more likely to be misclassified by parents as overweight as compared to younger children [29]. Huang et al. also observed that age was associated with parents’ misperception of children’s weight status [38]. In contrast, He and Evans did not find an association between child’s age and parents’ misperceptions of their child’s weight status [24].

A child’s sex is another factor that can affect the accuracy of parental perception of children’s weight status [20,23,24,35,42]. Similar to the results observed in this study, other authors reported that parents of overweight (overweight or obese) boys were more likely to incorrectly classify their son’s weight status [20,23,24,35]. These findings differ from a report by Maynard et al., where parents of children who were at risk for overweight were more likely to incorrectly classify their daughter compared to their sons [29]. Some studies did not find any association between a child’s sex and the misclassification of weight status [3,44]. Overall, it seems likely that parents may be more accepting of higher weight among their sons than among their daughters. It is possible that parents paid more attention to girls’ body image than the boys’ body image. Societal norms about the ideal weight for boys and girls may also have a role [24].

Some authors report that mothers are more accurate in assessing their child’s weight than fathers [28,50]. It was observed that parental misperception of weight status was associated with the respondent being the father in one study [40]. Similar to the work of De La O et al., our study did not show any association between misclassification of weight status and the responder [35].

Certain socioeconomic factors such as parental education, income level, and ethnicity could influence parental perception of children’s weight. We did not find a relationship between parental education and inaccurate perception of their child’s weight status, similar to what has been reported in other studies [3,18,24,35,44,51]. In some studies, lower educational status of the parents has been found to be associated with the parent’s inaccurate perception of their child’s weight status [40,45,52].

We found that non-Caucasian parents were more likely to misclassify their child’s weight status. Non-Caucasian children accounted for 8% of the total study population and of those, most reported being First Nations or Métis. Parental perception of child’s weight has been shown to vary with ethnic differences [24,45,53]. Doolen et al. reported that cultural influence was associated with parental perception of their child’s weight status [45]. He and Evans observed that more non-white parents were not able to accurately identify their children’s weight status [24]. Killion et al. has also shown that minority parents consistently underestimate the weight of their children [53]. In contrast, others have found no association between race/ethnicity and parental assessment of children’s weight status [23,29]. Further investigation is needed to understand the cultural aspects of body perception among non-Caucasians.

There are several strengths of this study. The study had sufficient statistical power to answer the objectives of the study. Information on additional factors, not reported in other studies were evaluated here, including type of household (single parent or two parents/partner), physical activity level, access to health care, fast food/drink intake, location of the home (farm/non-farm) and presence of any health conditions. We did not find any significant association between these factors and misclassification of parental perception of child’s weight status. Another advantage of this study was the availability of objective anthropometric measurements to evaluate parental reports of weight and height.

In this analysis, age and sex adjusted BMI categories are used to represent the weight status of the children. BMI is a simple, inexpensive measure of weight adjusted for height and it is a well-recognised measure of excess body weight [54]. Studies have shown that BMI categories correlate with body fat and health risks [55,56]. Factors such as age, sex, ethnicity, height and level of sexual maturation can influence the relationship between BMI and body fat among children [57,58,59] and with the exception of level of sexual maturation, were considered in this study.

On the other hand, this study has limitations. Some of the predictors of inaccurate parents’ perception of their child’s weight status found in other studies were not evaluated in the current study. Neither objective measure nor subjective reports of parental weight and perinatal history were obtained. Higher maternal BMI and higher infant birth weight have been shown to associate with an underestimation of child weight [3]. The authors of some studies reported that overweight mothers underestimate their child’s weight status [23,24]. Agras et al. reported that parent’s weight status was the strongest predictor for childhood overweight [60]. In this study the parent who was more familiar with the child’s health was asked to complete the questionnaire. About 8% of responders were fathers and nearly 90% of responders were mothers. Therefore, this sample may not fully represent the paternal perception.

We found the weight status of boys was more likely to be misclassified by parents. We were unable to conduct a separate analysis to determine associated covariates (physical activity levels, eating habits) for boys and girls as we did not have a sufficient number of cases who were misclassified.

Both physical activity and fast food intake of children were based on self-reports by parents. The discrepancy between self-reported and measured physical activity have been examined by others [61,62]. Researchers reported response bias and less chance of identification of associations between outcome and self-reported variables of physical activity or food intake [63,64]. Objective measures of physical activity and use of food diaries would have been useful, however additional costs of such measures were beyond this study.

We selected certain schools from the four study quadrants of the SRHS [27]. Therefore, our findings from this study may not be seen as representative of all children in rural Saskatchewan. However, when compared to the overall children aged 6 to 14 years in the 2010 rural covered population of the Saskatchewan Health Registry, our sample had a similar distribution by sex, but had a greater proportion of 6 to 10 year olds, suggesting that the findings for boys and girls could be generalized to the rural population of Saskatchewan while any age related findings could not.

Our findings suggest that parents have a poor awareness of their child’s weight status when reporting weight status according to BMI cut-offs. This could be due to the less understanding of the meaning of BMI cut-offs and in particular, overweight status. Another reason for misclassifying weight status could be that parents are aware of their child’s weight, but due to social undesirability, they do not wish to label their children as overweight.

Parental reporting of child weight status has implications for public health policy. Efforts should focus on improving parent’s perception of their child’s weight status. Future research should focus on assessing longitudinal effects of parental misperceptions of weight status of children.

## 5. Conclusions

In conclusion, this study found that the prevalence of being overweight was high in this group of rural children. Parental perception of weight markedly underestimates the actual weight status of overweight/obese school-age children. Parents were more likely to misclassify their child’s weight status if the child was a boy and from a non-Caucasian ethnic background.

## Figures and Tables

**Table 1:** Characteristics of the study population *n* = 584 children described using frequencies (%), means (SD: standard deviation) and medians (IQR: inter quartile range).

Variable		Statistic
Age, in years (mean ± SD)		9.6 ± 2.2
Sex: *n* (%)	Male	307 (52.6)
	Female	277 (47.4)
Ethnic background: *n* (%)	Caucasian	528 (90.4)
	Non-Caucasian	46 (7.9)
	Missing	10 (1.7)
Responder: *n* (%)	Mother	523 (89.6)
	Father	48 (8.2)
	Both (Mother & Father)	5 (0.9)
	Missing	8 (1.4)
Responder’s highest education ^a^: *n* (%)	Grades 1–8	26 (4.5)
	Grade 12	176 (30.1)
	Technical School	214 (36.6)
	University Degree	150 (25.7)
	Missing	18 (3.1)
Type of household: *n* (%)	Single Parent Home	55 (9.4)
	Two Parent/Partner Home	524 (89.7)
	Missing	5 (0.9)
Ever experience any difficulties obtaining routine or on-going health care: *n* (%)	Yes	39 (6.7)
	No	539 (92.3)
	Missing	6 (1.0)
Over the past 7 days, number of days physically active at least 60 min:		
mean ± SD		5.2 ± 1.6
median (IQR)		5.0 (3.0)
Over a typical or usual week, number of days physically active at least 60 min:		
mean ± SD		5.2 ± 1.6
median (IQR)		5.0 (2.5)
Parent’s perception of child weight: *n* (%)	Underweight	35 (6.0)
	Just about the right weight	503 (86.1)
	Overweight	46 (7.9)
Clinically measured BMI categories: *n* (%)	Underweight	28 (4.8)
	Normal	401 (68.7)
	Overweight ^b^	155 (26.5)
Fast food/drinks ^c^ intake more than 1 per week: *n* (%)	Yes	339 (58.0)
	No	245 (42.0)
Home location: *n* (%)	Farm	256 (43.8)
	Non-farm	311 (53.3)
	Missing	17 (2.9)
Any health conditions ^d^: *n* (%)	Yes	471 (80.7)
	No	113 (19.3)

^a^ Responders were mother, father and both mother and father. When both were specified their highest education was considered; ^b^ Overweight and Obese categories were combined as “overweight”; ^c^ Fast food/drinks includes French fries, hamburgers, fast food, deep fried food, chicken nuggets, soft drinks or pops, potato chips; ^d^ Ever diagnosed with any health conditions includes wheeze, shortness of breath, asthma, hay fever, eczema, diabetes, arthritis, tonsillitis, ear infection, stomach reflex and any allergy.

**Table 2:** Association between actual measured weight status and parental perception and parental report of their child’s weight status.

	Child’s Actual Measured Weight Status			Total *n* (%)	N, Test Statistic * and *p*-value
	Underweight *n* (%)	Normal *n* (%)	Overweight ^c^ *n* (%)		
**Parental Perceptions** ^a^					
Underweight	**11 (39.3)**	24 (6.0)	0 (0.0)	35 (6.0)	*N* = 584;
Just about the right weight	17 (60.7)	**376 (93.8)**	110 (71.0)	503 (86.1)	χ^2^ = 189.9;
Overweight	0 (0.0)	1 (0.2)	**45 (29.0)**	46 (7.9)	*p* < 0.0001
**Parental report of child’s weight status** ^b^					
Underweight	**13 (59.1)**	33 (9.8)	0 (0.0)	46 (9.4)	*N* = 490;
Normal	7 (31.8)	**279 (83.0)**	30 (22.7)	316 (64.5)	χ^2^ = 308.6;
Overweight^c^	2 (9.1)	24 (7.1)	**102 (77.3)**	128 (26.1)	*p* < 0.0001

* Pearson Chi-squared test statistic and Fisher’s exact test *p* values were reported. Boldface values indicate the accuracy of the parent perception and the parental report of the child weight; ^a^ The accurate perception of weight status in the total sample was 74%; ^b^ Accurate weight status reported in the total sample was 80%; ^c^ Overweight and obese categories were combined as “overweight”.

**Table 3:** Comparison of parent reported and actual measured mean weight, height, calculated BMI stratified by sex.

Variable	Weight in kg	Height in cm	Body Mass Index kg/m^2^
Boys, mean ± SE			
Parent Reported	37.69 ± 0.87	141.19 ± 0.99	18.47 ± 0.22
Actual Measured	39.11 ± 0.94 **	142.75 ± 0.94 **	18.70 ± 0.23
Girls, mean ± SE			
Parent Reported	37.94 ± 0.78	141.60 ± 1.08	18.83 ± 0.31
Actual Measured	39.94 ± 0.87 *	143.19 ± 0.93 **	19.16 ± 0.25

** *p* < 0.0001, * *p* < 0.01; SE: standard error of mean; Comparisons of parent reported and actual measurements were made among boys and girls separately.

**Table 4:** Frequency of misclassification of parent’s perceived weight status, unadjusted and adjusted odds ratios (ORs) and 95% confidence intervals (95% CI) based on logistic regression on misclassification of BMI; *n* = 584 ^a^.

Variable	Misclassification		Unadjusted OR (95% CI)	Adjusted OR (95% CI)	*p*-value
	Yes	No			
**Child age**, mean ± SD	9.86 ± 2.17	9.47 ± 2.22	1.08(0.99, 1.18)	1.08 (0.99, 1.19)	0.07
**Child sex**, *n* (%)					
Male	88 (57.9)	219 (50.7)	1.36 (0.94, 1.98)	**1.58 (1.06, 2.35)**	**0.02**
Female	64 (42.1)	213 (49.3)	1.00	1.00	-
**Responder**, *n* (%)					
Mother	135 (90.0)	388 (91.1)	0.23 (0.04, 1.40)	0.19 (0.03, 1.19)	0.08
Father	12 (8.0)	36 (8.5)	0.22 (0.03, 1.49)	0.17 (0.02, 1.23)	0.08
Both mother & father	3 (2.0)	2 (0.5)	1.00	1.00	-
**Respondent’s highest education**, *n* (%)					
Grades 1–8	6 (4.1)	20 (4.8)	0.68 (0.26, 1.80)	0.60 (0.22, 1.67)	0.33
Grade 12	38 (25.9)	137 (32.8)	0.63 (0.38, 1.03)	0.65 (0.38, 1.09)	0.10
Technical School	57 (38.8)	157 (37.6)	0.80 (0.51, 1.27)	0.89 (0.56, 1.45)	0.67
University degree	46 (31.3)	104 (24.9)	1.00	1.00	-
**Ethnic Background**, *n* (%)					
Non-Caucasian	17 (11.3)	29 (6.8)	1.76 (0.94, 3.30)	**2.03 (1.00, 4.16)**	**0.05**
Caucasian	133 (88.7)	395 (93.2)	1.00	1.00	-
**Type of household**, *n* (%)					
Single parent home	17 (11.3)	38 (8.9)	1.32 (0.72, 2.41)	-	-
Two parent/partner home	134 (88.7)	390 (91.1)	1.00		
**Past 7 days, number of days physically active at least 60 min**, mean ± SD	4.99 ± 1.63	5.23 ± 1.61	0.92 (0.82, 1.03)	0.89 (0.79, 1.01)	0.07
**Difficulties obtaining routine or on-going health care**, *n* (%)					
Yes	5 (3.3)	34 (7.9)	0.40 (0.16, 1.05)	0.41 (0.15, 1.08)	0.07
No	145 (96.7)	394 (92.1)	1.00	1.00	-
**Fast food/drinks intake more than 1 per week**, *n* (%)					
Yes	90 (59.2)	249 (57.6)	1.07 (0.73, 1.55)	-	-
No	62 (40.8)	183 (42.4)	1.00		
**Home Location**, *n* (%)					
Farm	63 (42.3)	193 (46.2)	0.85 (0.58, 1.25)	-	-
Non-farm	86 (57.7)	225 (53.8)	1.00		
**Any Health Condition**, *n* (%)					
Yes	123 (80.9)	348 (80.6)	0.98 (0.61, 1.56)	-	-
No	29 (19.1)	84 (19.4)	1.00		

^a^ For some variables numbers will not add up to 584 due to missing values.

## Abbreviations

The following abbreviations are used in this manuscript:

BMI	Body mass index
OR	Odds ratio
CI	Confidence Interval
SD	Standard deviation
IQR	Inter quartile range
SE	Standard error of mean
SRHS	Saskatchewan Rural Health Study
